# 

**Published:** 1894-06-09

**Authors:** 


### SPECIAL HOSPITAL SUNDAY SUPPLEMENT.

CONTENTS.

**The Humanity of Medicine** - - - - - - - - 199
**Around the Hospitals** - - - - - - - - 200
**Sir Benjamin Ward Richardson's Researches** - - - 201
**Studies in Applied Sociology.** X.—How Workers Live - 202
**Men Who Have Made Moden Psychiatry** - - - - 203
**Some Aspects of Medical Science** - - - - - 204
**Notes and News** - - - - - - - - - 206
**Medical Progress and Hospital Clinics—**
Acute Intussusception. I. - - - - - - - 207
Observations Regarding Chronic Heart Disease Complicating Pregnancy and Labour - - - - - - - 208
Gonorrhœa and Its Effects - - - - - - - 210
Medical Progress.—Progress in Nervous Diseases - - 211
**Editor's Letter-Box** - - - - - - - - - 216
**Annotations.**—Sir Edward Sieveking on Out-patients—Dermatology and General Medicine—Small-pox and Vaccination at Leicester - - - - - - - - - - 205
**"The Hospital's" Special Hospital Sunday Supplement, 1894**—The Future of Voluntary Hospitals—Ex Nihilo Nihil Fit—Is a Catastrophe Impending?—Threatened Interests—Dare We Run the Risk?—Immeasurably Great—General, Special, Miscellaneous—Ninety Thousand Modern Miracles—A Department which Might Be Better—Priced and Priceless—The "Seven Circles"—Our Fathers Built: Shall we Destroy? —Unification — Constitutionalism — To Fair-minded Englishmen—Summary of Work Done in 1893 - - - - 215

### EXTRA SUPPLEMENT.—THE NURSING MIRROR.

**News from the Nursing World.**—Honourable Obligations—Princess Christian at Soho—A Prize Quilt—An English Heroine—Newton Abbot Registered Nurses—Poor and Helpless—Caretakers for Idiots—New Rules at Addenbrooke's Hospital—Popular Appointments—Baths in Asylums—Nursing at Hamburg—Sick Nurses in Denmark—Short Items - - xci

**On General Nursing.** XV.—Rheumatism (*continued*) - - xciii

**The Mechanism of Movement.** II.—The Skeleton - - xciv

**News from Melbourne** - - - - - - - - xcv

**Past and Present Workhouses** - - - - - - xcv

**Nurses as Stewardesses**, xcv; **English Nurses in Brazil** xcvi

**Workhouse Infirmary Nursing Association** - - - xcvii

**For Reading to the Sick** - - - - - - - xcviii

**Where to Go for a Holiday.**—The Pilgrims' Path - - xcviii

**Exhibitions and Entertainments** - - - - - xcviii

**The Muses' Looking-Glass—The Nurse Dolls** - - xcix

**Everybody's Opinion** - - - - - - - - c

**A Successful Cafe Chantant—Appointments** - - - c

